# Simultaneous Quantitative Live Cell Imaging of Multiple FRET-Based Biosensors

**DOI:** 10.1371/journal.pone.0061096

**Published:** 2013-04-16

**Authors:** Andrew Woehler

**Affiliations:** 1 Deutsche Forschungsgemeinschaft-Research Center for Nanoscopy and Molecular Physiology of the Brain, Göttingen, Germany; 2 Department of Membrane Biophysics, Max-Planck Institute for Biophysical Chemistry, Göttingen, Germany; University of California Irvine, United States of America

## Abstract

We have developed a novel method for multi-color spectral FRET analysis which is used to study a system of three independent FRET-based molecular sensors composed of the combinations of only three fluorescent proteins. This method is made possible by a novel routine for computing the 3-D excitation/emission spectral fingerprint of FRET from reference measurements of the donor and acceptor alone. By unmixing the 3D spectrum of the FRET sample, the total relative concentrations of the fluorophores and their scaled FRET efficiencies are directly measured, from which apparent FRET efficiencies can be computed. If the FRET sample is composed of intramolecular FRET sensors it is possible to determine the total relative concentration of the sensors and then estimate absolute FRET efficiency of each sensor. Using multiple tandem constructs with fixed FRET efficiency as well as FRET-based calcium sensors with novel fluorescent protein combinations we demonstrate that the computed FRET efficiencies are accurate and changes in these quantities occur without crosstalk. We provide an example of this method’s potential by demonstrating simultaneous imaging of spatially colocalized changes in [Ca^2+^], [cAMP], and PKA activity.

## Introduction

Molecular biosensors based on intra-molecular FRET have become indispensible tools for monitoring the spatial and temporal regulation of signaling processes in living tissue. A number of FRET-based genetically encoded sensors quantifying second messenger concentration, phosphorylation state, and GTPase activity have been developed and improved throughout the last decade [1]. Although these sensors have already proven to be invaluable at probing individual processes [2], it is becoming increasingly apparent that in order to better understand the complex interaction networks responsible for signal transduction that the ability to monitor the activity and spatial localization of multiple processes simultaneously is required [3].

Commonly, individual processes are examined sequentially in a number of measurements from different samples, in which common ‘fiduciary’ events exists [4]. Information about the individual processes is then combined to build a broader picture of the signaling network. Such approaches, termed computational multiplexing, have been applied in reconstructing the spatiotemporal relationship of signaling events measured with respect to, for example, the timing of ligand application, changes in membrane potential, or changes in membrane shape [5], [6]. Useful endogenous fiduciary events do not exist for all processes and exogenous events imposed upon the system often perturb the normal dynamics one wishes to investigate. Furthermore, the interdependence of seemingly stochastic events is an interesting feature and by its nature cannot be studied by computation multiplexing.

To address the limitations of computational multiplexing, advances have been made in multiplexing measurements experimentally. In the past, the use of genetically encoded FRET-based sensors in parallel has been limited by the cross excitation and emission bleed-through of the fluorescent proteins available, such that quantification of FRET without crosstalk has been a major challenge. One approach to side-step this hindrance has come with the development of novel fluorescent proteins, generally with excitation and emission peaks separated from those of CFP and YFP. Some of these have especially large stokes shifts, which allow for orthogonal wavelength measurements. When combined with four color widefield imaging, these approaches have allowed users to monitor two processes simultaneously [7], [8].

In the following we introduce a novel method for FRET analysis based on linear unmixing of 3D excitation/emission fingerprints. By computing the spectral fingerprint of FRET from reference measurements, the total relative concentrations of each fluorophores and scaled FRET efficiencies can be directly unmixed from the excitation/emission spectrum of a FRET sample without the need for additional corrections for excitation crosstalk and emission bleed-through. We use this method to separate the FRET efficiencies of three different sensors each composed of two out of a total of three different fluorophores. The full utility of this method is then demonstrated by simultaneously imaging spatially colocalized changes in [Ca^2+^], [cAMP], and PKA activity.

## Results

### Theory

This method is based on luxFRET, which was developed for analyzing conventional single donor/single acceptor systems [9]. In luxFRET fluorescence emission is measured over a broad spectral range and donor/acceptor fluorescence contributions are separated through spectral decomposition. Rather than filtering the signal to maximize the specificity of an emission channel to a select fluorophore, spectral overlap is welcomed in order to maximize photon collection, with bleed-through negated through linear unmixing. The extension of luxFRET presented here differs slightly from the previous implementation not only by accounting for an additional interacting fluorescent species but also in the approach to spectral decomposition. In Wlodarczyk et al 2008 [9], linear unmixing of the FRET sample was performed separately on measurements at two different excitation wavelengths. Unmixing provided apparent donor and acceptor concentrations, which were then used to compute the total donor and acceptor concentrations, relative to the reference samples, as well as the apparent FRET efficiency. The extended method presented here requires the same reference measurements and calibration terms as luxFRET but uses them to define the spectral fingerprint of FRET between a given fluorophore pair in a 3D excitation-emission fluorescence spectrum. It is then possible to unmix the spectrum of a FRET sample using the fluorophores and FRET spectra and directly compute the relative total concentrations and apparent FRET efficiencies.

We begin by considering the fluorescence from a sample expressing three independent intramolecular FRET sensors: a CFP/YFP labeled FRET sensor at a concentration of [CY] with FRET efficiency E_CY_, a CFP/RFP labeled FRET sensor at a concentration of [CR] with FRET efficiency E_CR_, and a YFP/RFP labeled FRET sensor at a concentration of [YR] with FRET efficiency E_YR_. The total fluorescence is a linear combination of these terms such that the emission from CFP, YFP, and RFP can be written as
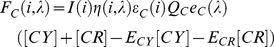
(1)

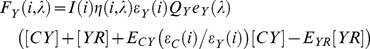
(2)

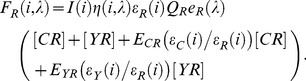
(3)



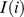
 is the relative excitation intensity at wavelength *i* (i.e. 1∶ 430 nm, 2∶ 500 nm, or 3∶ 575 m). 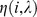 represents the spectral detection efficiency of the instrument used and may differ between excitation wavelengths if different filters are used. 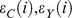 and 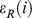 are the extinction coefficients of donor and acceptor at the three respective excitation wavelengths. 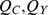
and 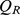
are the quantum yields of the CFP variant mTq2 (0.93 [10]), the YFP variant cpVenus (0.56 [11]) and the red fluorescent protein mCherry (0.22 [12]), respectively, and 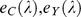 and 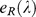 are the probability distributions of emission of the three fluorescent proteins (unit area normalized emission spectra).

By performing reference measurements of each of the separate fluorophores with the same settings as used in the FRET measurement we can quantify the fluorophore specific parameters scaled by the reference concentration. For example, the fluorescence measured from a sample expressing only CFP can be written as
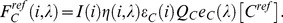
(4)


Using the reference measurements to eliminate the fluorophores-specific parameters we can combine equations 1–3 to define the total fluorescence from a CFP/YFP/RFP FRET sample as
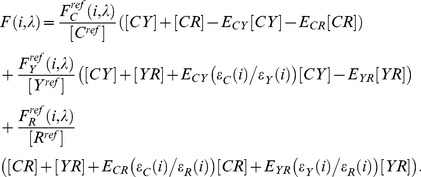
(5)


**Figure 1:**
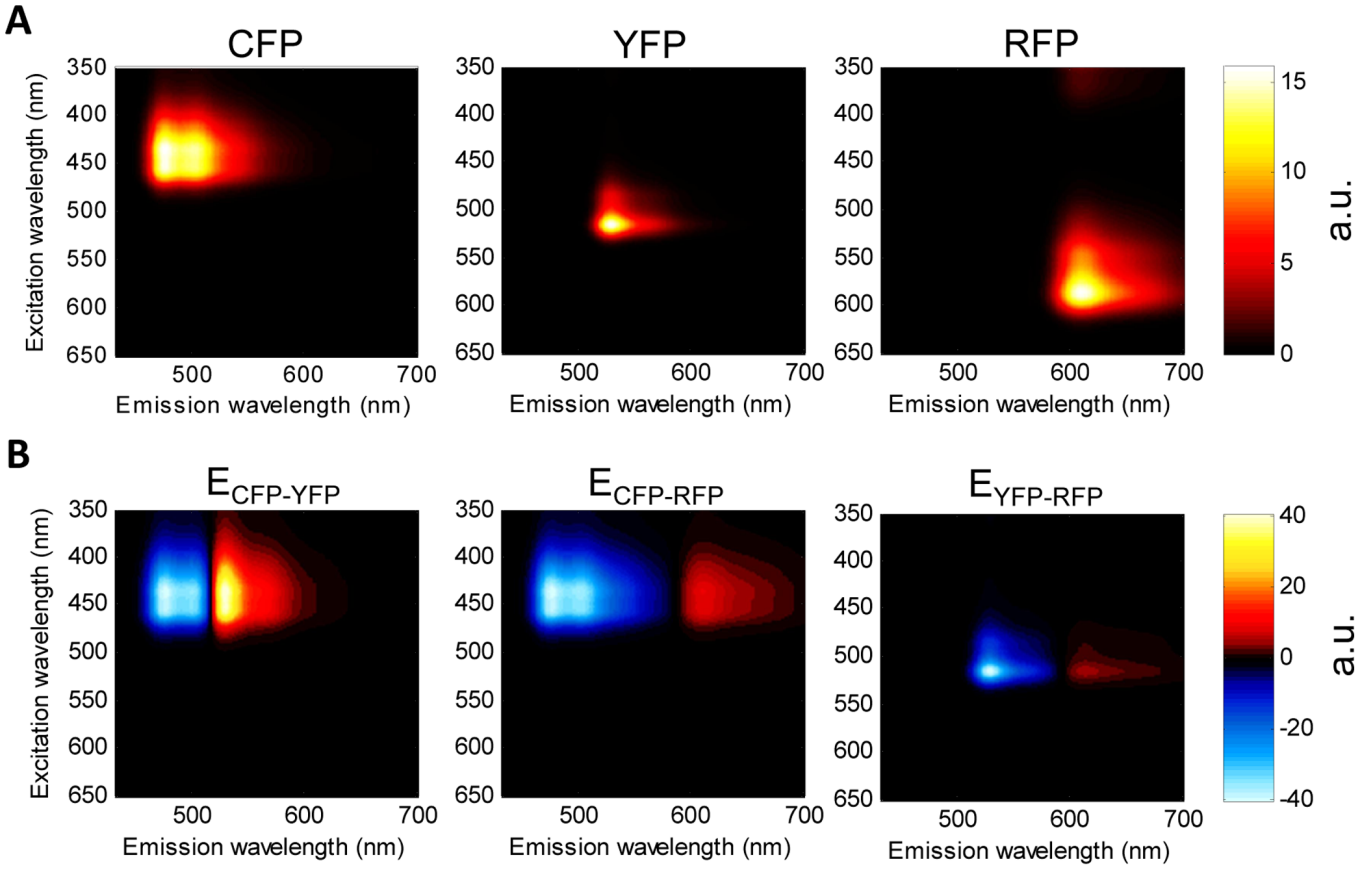
FRET spectral fingerprints. **A)** 3D excitation/emission reference spectra for mTurquoise2 (CFP), cpVenus (YFP), and mCherry (RFP), respectively from left to right, were constructed from single high resolution excitation and emission spectra. B) Emission fingerprints (3D excitation/emission spectra) of FRET were computed by subtracting the donor reference spectrum from the product of the appropriate calibration function, 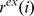, and the acceptor reference spectrum. Shown from left to right are the spectral fingerprints for energy transfer from mTq2 to cpVenus, mTq2 to mCherry, and cpVenus to mCherry.

Rearranging this equation we arrive at.
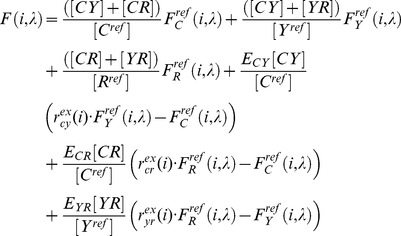
(6)where 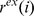 are calibration functions that represent the relative excitability of the two noted fluorophores at excitation wavelength *i*, scaled by the appropriate reference concentration ratio [9]. For example, for CFP and YFP,



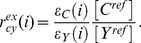
(7)The first three terms of equation 6 (first line) represent the fluorescence that would be observed if there were no FRET. The last three terms in equation 6, that combine the calibration functions with reference spectra, represent the quenching and sensitization that occur with FRET. For example in the case of FRET between CFP and YFP, the fourth term subtracts fluorescence with the spectral characteristics of CFP, indicating donor quenching, while adding fluorescence with the excitation characteristics of CFP but the emission characteristics of YFP, indicating sensitized emission. Considering equations 4 (for YFP) and 7 it becomes apparent that by multiplying 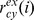 by the YFP reference spectrum 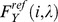, 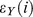 is eliminated and replaced with 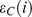
changing the excitation spectral shape to that of CFP. While the emission shape of YFP is maintained, the reference concentration ratio included in 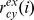 (eq. 7) eliminates 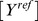 from the term and scales the amplitude of emission with respect to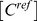. Figure 1 panel A illustrate the spectral fingerprint of CFP (mTq2), YFP (cpVenus), and RFP (mCherry) as computed and interpolated from single excitation and emission spectra measured on a spectrofluorometer. While panel B represent the FRET spectral fingerprints between CFP-YFP, CFP-RFP, and YFP-RFP computed from the measured reference spectra and the calibration functions computed as in Wlodarczyk et al 2008 [9]. By unmixing the spectral fingerprint of a sample of interacting CFP, YFP, and RFP using these reference spectra and the computed FRET spectral fingerprints, the coefficients in equation 6 that represent total relative concentrations of CFP, YFP, and RFP as well as the scaled FRET efficiencies are directly quantified. Apparent FRET efficiencies can then be computed by dividing unmixed scaled FRET efficiencies by the appropriate unmixed total relative concentration to eliminate scaling to the reference concentrations.

In the case that each fluorophore species present in the sample is used in two independent constructs, the measured total concentration of a fluorophore is the sum of the concentrations of these sensors. The measured apparent FRET efficiency is thus sensitive to the relative abundance of sensors in the sample. In order to estimate the absolute FRET efficiency to compare values between samples with different relative expression, the relative concentrations of the individual sensors must be determined. By performing the analysis introduced above and computing the acceptor to donor ratio for each FRET sensor of known A:D stoichiometry, typically 1∶1 for most sensors, the respective reference concentration ratios can be determined. For example the ratio of YFP total relative concentration to CFP total relative concentration, 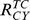, measured from a 1∶1 CFP/YFP tandem construct provides the ratio of reference concentrations 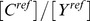:
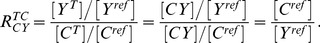
(8)


The measured total relative concentration of a given fluorophore in the system of three sensors is the sum of the concentrations of the constructs in which it is utilized. This allows a system of linear equations to be constructed:
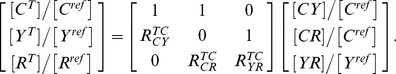
(9)


Here, the reference concentration ratios, as determined by equation 8, are required to appropriately change the denominators (reference concentrations) in some terms, so they may be summed. Solving then for the sensor concentration yields 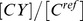, 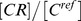, and 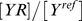. These recovered sensor abundances are then used to eliminate all concentration dependent factors in the scaled FRET efficiencies recovered from the spectral decomposition.

### Spectral Imaging and FRET Analysis

**Figure 2:**
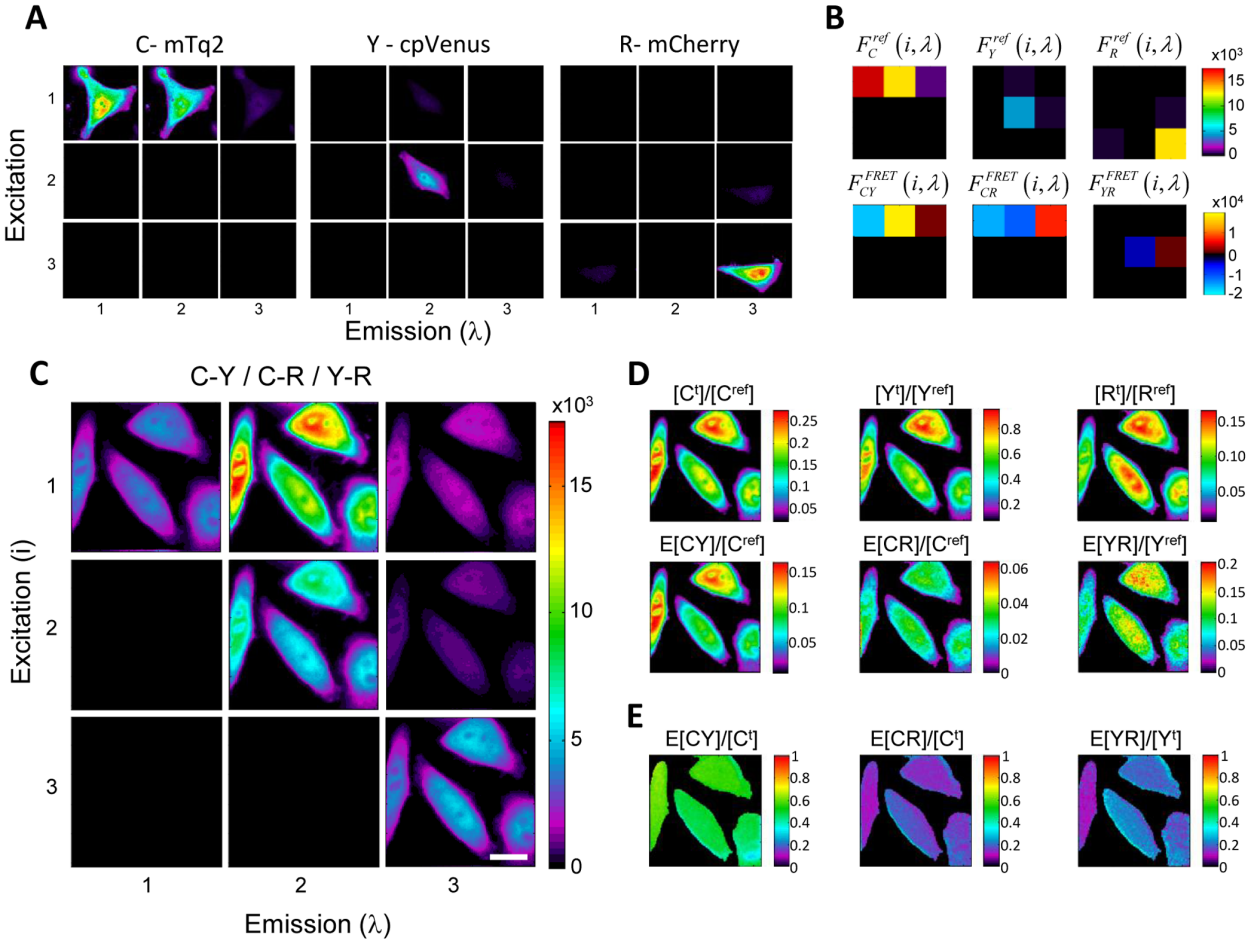
Spectral imaging and FRET analysis. **A)** Spectral images of HeLa cells expressing mTq2, cpVenus and mCherry are presented respectively from left to right. Excitation channels 1,2, and 3 correspond to 430 nm, 500 nm, and 575 nm, respectively, while emission channels 1,2, and 3 correspond to 455–485 nm, 520–550 nm, and 600–670 nm, respectively. **B)** Fluorescence sampled from the reference images is used to create reference spectra illustrated in the top row for mTq2, cpVenus, and mCherry, respectively, with the x and y axis representing excitation and emission channels. The spectral fingerprints of FRET are computed using the appropriate reference spectra and calibration functions for transfer from mTq2 to cpVenus, mTq2 to mCherry, and cpVenus to mCherry, bottom row, left to right respectively. **C)** Spectral imaging of multiple FRET processes is performed in HeLa cells coexpressing mTq2-15AA-cpVenus, mTq2-15AA-mCherry, and mCherry-15AA-cpVenus. **D)** Unmixing the FRET sample using the reference and FRET spectral fingerprints directly quantifies the total relative concentrations (top row) and scaled FRET efficiencies (bottom row). **E)** Apparent FRET efficiencies can be computed by dividing the scaled FRET efficiencies by the appropriate total relative concentration. Scale bar represents 20 um.

Rather than utilizing high resolution excitation/emission spectra as illustrated in Fig. 1, compromises must be made when applying this method to microscopy. Here we demonstrate the application of this method to spectral imaging using the minimal set of required measurements. Using only three excitation wavelengths and three fixed emission channels provides six measurements that can be used to define six quantities. Reference measurements of samples expressing CFP (mTq2), YFP (cpVenus), and RFP (mCherry) alone (Fig. 2A) as well as measurements of the FRET sample (Fig. 2C) are performed with the same settings as described in Methods. Briefly, for each sequential excitation wavelength (i: 430 nm, 500 nm, 575 m) fluorescence emission is split into three channels (λ: 455–485 nm, 520–550 nm, 600–670 nm) and detected simultaneously in three quadrants of a single EMCCD camera. After separating and spatially aligning background subtracted images of each of the reference samples, regions of interest (ROIs) were defined and mean fluorescence sampled. This resulted in the 3D excitation/emission reference spectra illustrated in Figure 2 panel B top row. These spectra are analogous to those illustrated in Figure 1, only measured from single cells at lower spectral resolution. These spectra are then used, along with quantum efficiencies and sampled unit-normalized spectra to define the calibration functions 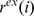 as described in Wlodarczyk et al 2008 [9], with 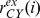 = [65.19, 0.01, 1.15] 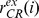 = [135.09, 0.01, 3.9E−4] 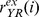 = [2.16, 1.96, 3.4E−4].

To construct the FRET spectral fingerprints for each fluorophores pair, the donor reference spectrum is subtracted from the product of the calibration function and the acceptor reference spectrum (Fig. 2B bottom row). This set of reference spectra is then used to unmix the spectra of each pixel of an image measured from a FRET sample expressing CFP-15AA-YFP, CFP-15AA-RFP, and YFP-15AA-RFP FRET constructs (Fig. 2C). The linear unmixing results in spatial maps of the total relative concentrations of CFP, YFP and RFP present in the sample as well as images of scaled FRET efficiencies (Fig. 2D). By dividing the scaled FRET efficiencies by the appropriate total relative concentration images of apparent FRET efficiencies are computed (Fig. 2E).

Apparent FRET efficiencies are dependent on the relative abundance of donor participating in a given FRET complex and will thus vary from sample to sample due to differences in relative expression of the constructs. To compare FRET values between samples, the relative abundance of sensors must be tightly controlled or the absolute FRET efficiency must be estimated. Using equation 9 and the reference concentration ratios, measured from the total relative concentrations described above and equation 8, the total relative sensor concentrations can be quantified. Dividing the scaled FRET efficiencies by the appropriate total relative sensor concentration eliminates all concentration dependent terms and estimates absolute FRET efficiency.

**Figure 3:**
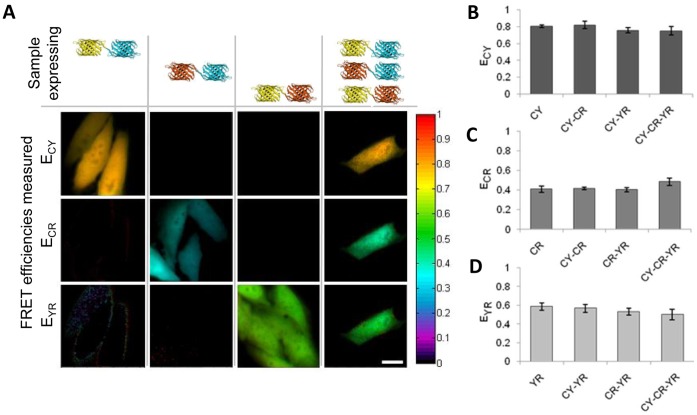
Separating FRET efficiencies. **A)** Selective FRET efficiencies are measured in HeLa cells selectively expressing mTq2-15AA-cpVenus, mTq2-15AA-mCherry, and mCherry-15AA-cpVenus. All three FRET efficiencies are measured in HeLa cells expressing all three constructs. **B)** FRET from mTq2 to cpVenus is accurately reported in samples selectively expressing the CY construct as well when coexpressing this construct along with CR, YR and CR/YR together. **C)** FRET from mTq2 to mCherry is accurately reported in samples selectively expressing the CR construct as well when coexpressing this construct along with CY, YR and CY/YR together. **D)** FRET from cpVenus to mCherry is accurately reported in samples selectively expressing the YR construct as well when coexpressing this construct along with CY, CR and CY/CR together. Images shaded by fluorescence intensity. Error bars represent +/− one standard deviation. Scale bar represents 20 um.

To evaluate whether three absolute FRET efficiencies could be estimated reliably from the same sample, the three tandem constructs described above were expressed separately in a set of samples as well as together in different combinations. For each of the singularly expressing samples the respective FRET efficiency is measured reliably (Fig. 3). Often, however, in cases that a given construct is not present in a sample, the measured scaled FRET efficiency and total relative concentration are small but nonzero. Dividing these values can result in noise with a mean that often is very small E <0.05 or very large E>>1, but in rare cases maybe be interpreted as FRET (Fig. 3A *E_CR_* and *E_YR_* for CY sample). This artifact can be greatly reduced through thresholding of the image based on absolute fluorescence intensity as well as the amplitude of unmixed quantities. Figure 3A illustrates the selective quantification of FRET efficiencies in samples expressing single constructs as well as quantification of comparable efficiencies in a sample expressing all three constructs. Samples expressing only the CY tandem construct measured only E_CY_ equal to 0.804+/−0.016 (n = 16). Whereas in samples expressing the CY construct in presence of CR, YR and both CR and YR, ECY was measured at 0.817+/−0.044 (n = 14), 0.756+/−0.032 (n = 15), and 0.750+/−0.050 (n = 13), respectively (Fig. 3B). For samples expressing CR alone E_CR_ equaled 0.409+/−0.034 (n = 11), or in presence of CY, YR, and both CY and YR, E_CR_ was measured as 0.417+/−0.013 (n = 14), 0.404+/−0.021 (n = 12), 0.485+/−0.037 (n = 13), respectively (Fig. 3C). Samples expressing YR alone, in presence of CY, CR, and both CY and CR, EYR was measured as 0.587+/−0.041 (n = 16), 0.567+/−0.043 (n = 15), 0.532+/−0.036 (n = 12), and 0.503+/−0.056 (n = 13), respectively (Fig. 3D). Although the values for the individual FRET efficiencies are comparable between samples there does seem to be a slight trend resulting in a reduced measure of ECY in presence of YR and CR/YR, an enhancement of ECR in presence of CY/YR (apparent in Fig. 1A as well) and a reduction of YR when in the presence of CR and CY/CR.

### Measuring Selective Changes in FRET

Often noise is the limiting factor in the utility of FRET measurements. This noise originates as shot noise and is less than favorably propagated through the analysis required to quantify FRET efficiency [13]. Another source of error arises from the uncertainty created by overlapping emission spectra [14]. Although this error can be minimized by filtering the fluorescence into channels that maximize the selectivity to a given fluorophores, we have previously shown that it is in fact advantageous to collect photons from overlapping regions and to separate them through linear unmixing [15]. In the method present here the error resulting from overlapping emission spectra is compounded because not only are we separating fluorescence from fluorophores with partially overlapping emission spectra but also separate fluorescence from the same fluorophore from two different sources. This results in a decrease in the amplitude of relative changes in fluorescence intensity that are used to quantify FRET and increased noise.

**Figure 4:**
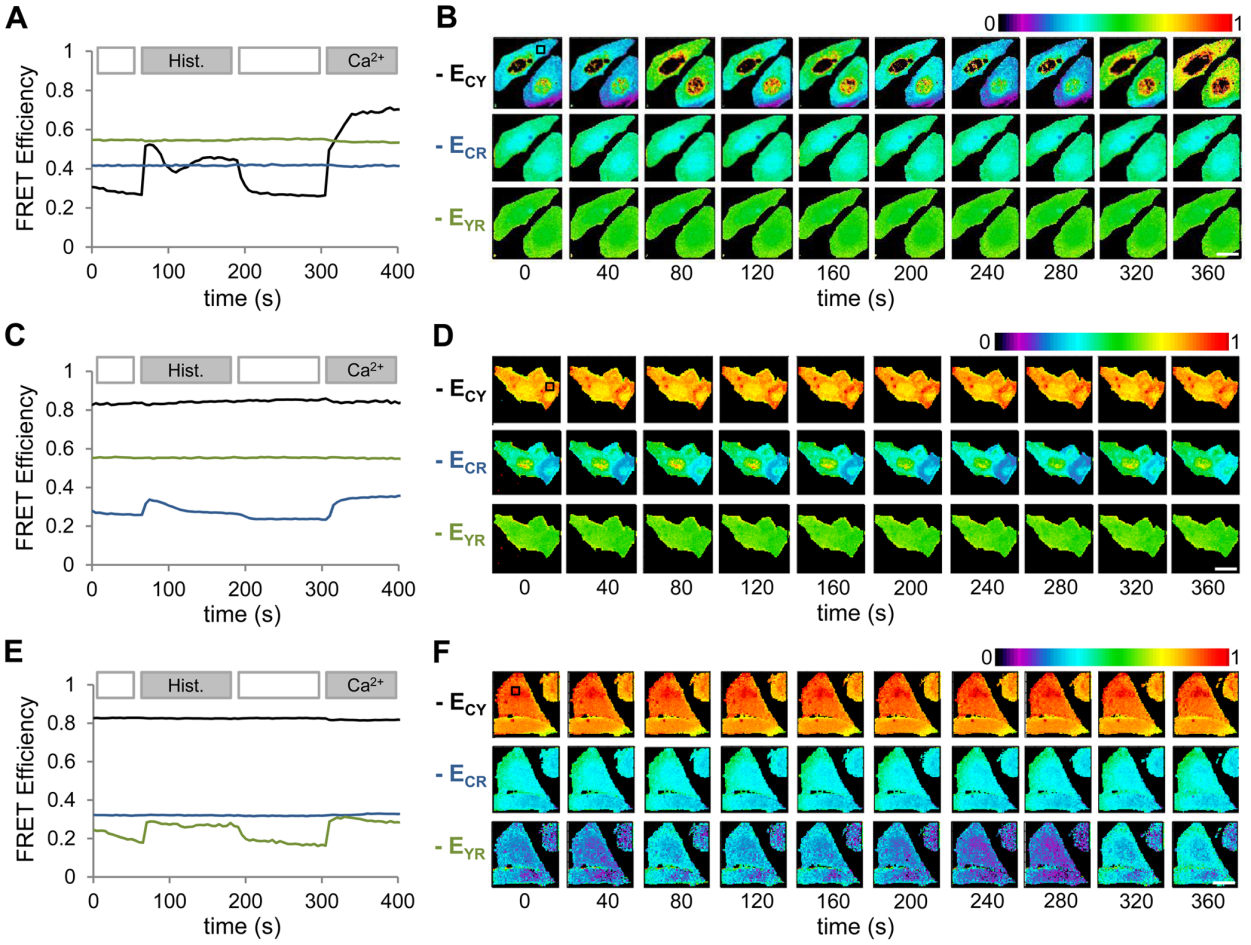
Measuring selective changes in FRET. **A)** Three FRET efficiencies are measured from HeLa cells expressing mTq2-TN-XXL-cpVenus, mTq2-15AA-mCherry, and mCherry-15AA-cpVenus. Upon application of 10 uM Histamine at 60 seconds and increase in *E_CY_* (black trace) is measured while *E_CR_* (blue trace) and *E_YR_* (green trace) remain constant. At 180 seconds histamine is washed out and *E_CY_* returns to baseline. At 300 seconds 10 uM ionomycin/5 mM Ca^2+^ was applied to saturate *E_CY_*. **B)** Spatial maps of E_CY_, E_CR_ and E_YR_ measured from two HeLa cells are illustrated. The dark spots surrounded by seemingly high FRET efficiency in the E_CY_ images result from the nuclear exclusion of the calcium sensor and low level of fluorescence in these regions. Further intensity based thresholding can eliminate such artifacts. **C,D)** Traces of mean FRET efficiency and spatial maps are shown for cells expressing mTq2-15AA-cpVenus, mTq2-TN-XXL-mCherry, and mCherry-15AA-cpVenus. **E,F)** Traces of mean FRET efficiency and spatial maps are shown for cells expressing mTq2-15AA-cpVenus, mTq2-15AA-mCherry, and mCherry-TN-XXL-cpVenus. All traces and images are representative of three or more measurements. Scale bar represents 20 um.

To evaluate whether changes in the FRET state of a sensor could be reliably measured while in presence of additional FRET constructs, the fluorescent proteins of a troponin-C based FRET sensor, TN-XXL [16], were swapped with the different combinations of mTq2, cpVenus, and mCherry. When the mTq2/cpVenus labeled sensor, denoted from now as CY-Ca^2+^, was expressed along with CR and YR tandem constructs in HeLa cells(Fig. 4A), an initial E_CY_ of approximately 0.3 was measured along with E_CR_ and E_YR_ values comparable to those in Fig. 3. Images were acquired at 5 second intervals and upon activation of the Gq signaling pathway by application of 10 uM histamine at 60 seconds a sharp increase in *E_CY_* was measured. At 180 seconds, *E_CY_* decreases upon wash out of histamine and then is saturated at ∼0.7 by application of 10 uM ionomycin with 5 mM Ca^2+^ at 300 seconds while *E_CR_* and *E_YR_* remain constant.

Similar measurements were performed with the CR-Ca^2+^ and YR-Ca^2+^ sensors expressed with corresponding tandem constructs (Fig. 4C–F). While the absolute change in FRET efficiency was not as large as for the CY-Ca^2+^ sensor, the changes were still easily resolved above noise. In addition to evaluating the suitability of this method to measure FRET changes in presence of multiple constructs, these measurements also indicate that the measured FRET changes are selective and that little or no crosstalk occurs between the measured FRET efficiencies.

### Quantifying Three Processes Simultaneously

**Figure 5:**
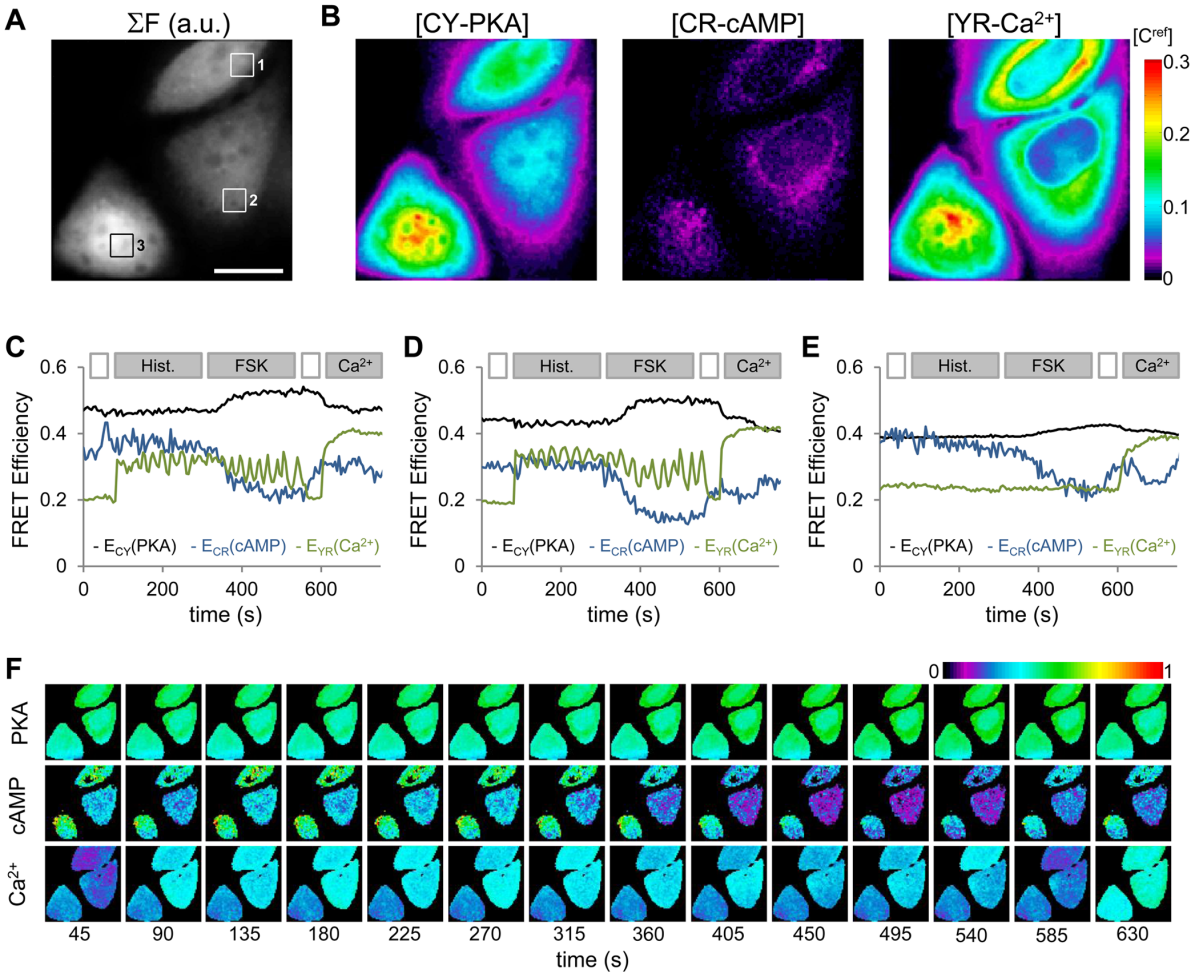
Simultaneous measurements of [Ca^2+^], [cAMP], and PKA activity. **A)** The summed fluorescence from all excitation/emission channels from the first acquisition of a spectral imaging time series of three HeLa cells coexpressing mTq2-AKAR-cpVenus (CY-PKA), mTq2-Epac-mCherry (CR-cAMP), and mCherry-TN-XXL-cpVenus (YR-Ca^2+^) is illustrated. Three regions of interest from which fluorescence is sampled for time series analysis are indicated. **B)** The unmixed spatial distributions of the CY-PKA, CR-cAMP, and YR-Ca^2+^ concentrations relative to the mTq2 reference sample concentration, [C^ref^], indicate relatively low [CR-cAMP] and nuclear exclusion of YR-Ca^2+^. **C–E)** Three FRET efficiencies are measured from each of the HeLa cells through sampling and subsequent analysis of the raw fluorescence from the ROIs 1–3 indicated in panel A, respectively. Upon application of 10 uM Histamine at 60 seconds and increase in *E_YR_* (green trace) is measured while *E_CY_* (black trace) and *E_CR_* (blue trace) remain constant. At 300 seconds 25 uM forskolin/100 uM IMBX was applied to increase [cAMP], indicated by the decrease in E_CR_, and activate PKA, indicated by the increase in E_CY_. At 540 seconds histamine/forskolin/IMBX is washed out and at 600 seconds calcium is elevated through the application of 10 uM ionomycin in 5 mM CaCl_2_ Ringer’s solution. **F)** Spatial maps of E_CY_, E_CR_ and E_YR_ measured from three HeLa cells are illustrated. All traces and images are representative of three or more measurements. Scale bar represents 20 um.

To demonstrate the full potential of this method we sought to measure three separate processes simultaneously. The fluorescent proteins of the PKA sensor AKAR3 [17] and an Epac1 based cAMP sensor [18] were exchanged for mTq2/cpVenus and mTq2/mCherry, respectively. HeLa cells were cotransfected with plasmids encoding CY-PKA, CR-cAMP and the YR-Ca^2+^ sensor described above (Fig. 5 A–B). Similar to the protocol outline above 10 uM histamine was applied at 60 seconds and a sharp increase and persistent oscillations are measured in the *E_YR_* in two of the three cells illustrated in Figure 5A, with no apparent change in *E_CY_* or *E_CR_* (Fig. 5C–E corresponding to ROI 1–3 in Fig. 5A, respectively). Upon activation of adenylate cyclase and inhibition of phosophodiesterates through the application of 25 uM forskolin/100 uM IMBX at 300 seconds a gradual decrease in *E_CR_* is measured indicating an increase in cAMP. After a short delay an increase in *E_CY_* is also measured indicating PKA activity. At 540 sec the bath solution is exchanged to wash out the histamine, forskolin and IMBX. The calcium oscillations cease and *E_YR_* drops to its original value. The washout of forskolin and IMBX decreases cAMP concentration resulting in an increase in *E_CR_* which is again is followed by a corresponding change in PKA activity. At 600 seconds 10 uM ionomycin with 5 mM Ca^2+^ is applied, increasing calcium and saturating the FRET efficiency of the calcium sensor while cAMP and PKA levels continue to return to baseline levels.

## Discussion

The ability to quantify the interaction of multiple proteins or measure multiple features of the intracellular environment simultaneously is necessary for continued progress in untangling the complex signaling networks that govern cellular behavior. We have introduced a novel method for three color FRET analysis that allows for just that. We have provided a theoretical framework under which multiple FRET efficiencies may be measured. With this we demonstrate the ability to quantify the FRET efficiencies and total relative concentrations of three independent FRET-sensors composed of the combinations of only three different fluorophores. The analysis has been streamlined compared to that of similar methods applied to two fluorophores systems [9], [19], [20], such that unmixing the 3D excitation/emission spectrum of a three fluorophores FRET sample of results in 6 unmixed quantities. Three of these represent the total relative concentrations. The other three quantities, when divided by the appropriate total relative concentration, represent the apparent FRET efficiencies. Through measurements of three fixed FRET efficiency tandem constructs expressed alone and together in different combinations we demonstrated that this method accurately quantifies FRET efficiencies in the presence of multiple constructs.

In the most extreme case of the coexpression of all constructs, not only is the standard deviation in the measured FRET efficiency slightly greater but the mean values seem to indicate some biasing. A major problem that plagues all fluorescence based quantification methods is incomplete labeling. In the case of genetically encoded tandem FP sensors, the existence of dark FPs due to maturation problems and photobleaching results in the existence apparently ‘free’ fluorescent species in the sample. Some estimates for the functional probability of FPs are as low as 0.80 [21]. The system of equations we developed to solve for the sensor concentrations does not account for the possibility of incomplete labeling. Including apparently free species would result in an underdetermined system of equations. The biasing seen in the triple coexpression could result from the system forcing fluorescence from ‘free’ species into the fixed stoichiometry constraints. For example, mCherry is the slowest to mature of the fluorescent proteins used and may have a lower steady state functional probability. This could result in apparently free mTq2 and cpVenus in the sample. The algorithm would suggest an increased apparent [CY]/[C^ref^]. With the apparent free species not contributing to FRET the unmixed E_CY_[CY]/[C^ref^] value would not be influenced, however by dividing the former by the latter E_CY_ would be underestimated. Whereas in the samples expressing only the CR construct the free mTq2 species may still force the system to overestimate [CR]/[C^ref^] resulting in decreased E_CR_. When coexpressed with the other sensors, the free species may be better fit with the other sensor concentrations, decreasing the apparent [CR]/[C^ref^] and increasing the estimated E_CR_. No ensemble level FRET method is unaffected by incomplete labeling and it is reasonable to expected the effects to be compounded as the complexity of the system is increased. As in the implementation of all quantitative fluorescence methods, users should take care in their interpretations of the absolute values measured.

To demonstrate the application of this method to dynamic measurements of intracellular processes, the fluorescent proteins used in three popular FRET sensors for calcium, cAMP, and PKA were exchanged with different combinations of the same cyan, yellow and red fluorescent proteins. To evaluate the fidelity of separating FRET efficiencies in the presence of dynamic changes in FRET we performed measurements of live cells coexpressing the different combinations of two fixed-FRET tandem constructs together with one calcium sensor. In these measurements the FRET efficiency of the fixed-FRET tandem constructs remain unchanged over time, while the efficiency of the calcium sensor is modulated through application of histamine and ionomycin/calcium to the cells and easily resolved above noise. We then demonstrated the full utility of this method by coexpressing three different sensors in the same cell and simultaneously measured dynamic changes in [Ca^2+^], [cAMP], and PKA activity.

This is not the first study in which FRET has been measured in a three color system. In addition to multiple studies utilizing three or more colors to study protein complex formation and conformational changes with single molecule measurements [22]–[25], others have performed spectral FRET measurements of fluorescent protein ensembles somewhat similar to the measurements present here [26], [27]. In the first of these studies, Gelperin and colleagues introduced ‘3-FRET’ to investigate the interaction of epidermal growth factor receptor (EGFR) with the adaptor protein Grb2 and the tyrosine phosphoprotein Cbl. They qualitatively identified interaction of these proteins labeled with CFP, YFP, and mRFP by measuring crosstalk and bleed-through corrected intensities of sensitized emission (equivalent to *nF* introduced in Youvan et al 1997 [28]) for each FRET pair. This quantity will change with FRET efficiency; however, it will also vary with absolute donor and acceptor concentrations. The authors then proceeded to estimate ‘*E*’ from *nF* through the use of *G-factors* for each FRET pair as suggested in Gordon et al 1998 [29]. This study introduced an additional approach that involves photo-bleaching the most red-shifted FP, mRFP in this case, measuring FRET from the donor de-quenching and then proceeding with 2-color FRET analysis.

Sun et al 2010 [27] used a three FP system to investigate the interaction of the dimeric transcription factor C/EBPα with the heterochromatin protein 1α (HP1α). This investigation begins with the presentation a model that extends the traditional single distance model for an additional fluorophore that acts as an acceptor to both the traditional donor and acceptor [30], [31]. They then derive a set of equations to evaluate the system that are similar to those used by Gelperin and colleagues. Similarly, these equations address the interdependence of FRET values when multiple acceptors are present, however using a different notation. Both of these approaches require measurements to correct for excitation crosstalk and emission bleed-through and rely on the use of extensive equations. While these equations are not technically difficult to implement, it is not trivial to follow the physical meaning of terms and operations. Additionally, when investigating intermolecular FRET, as in both of these studies, the fraction of interacting species is often the quantity that changes, which is not considered in the single distance model from which the analysis is derived [32]. Therefore it is difficult to interpret whether the derived apparent FRET efficiencies are sensitive to free donor or acceptor molecules and what exactly differences in FRET efficiency represent. While the method presented here does not aim at addressing the problem of interdependent or intermolecular FRET it nonetheless could be applied to such studies. Not only is this extension of luxFRET easier to apply, with no corrections need for excitation crosstalk or emission bleedthrough, it provides quantities with unambiguous physical meaning: total relative concentrations and apparent FRET efficiencies.

To our knowledge this is the first implementation of quantitative spectral FRET to a 3-color system that measures multiple dynamic processes. Other approaches, however, have found use in multiplexing fluorescence measurements of signaling processes. Typically these methods rely on intensity-based organic calcium sensors combined with ratiometric FRET measurements of genetically encoded sensors [33]. Alternatively, methods that use ratiometric FRET measurements of two such sensors with orthogonal excitation/emission wavelengths have also been developed [7], [26]. In one of the most notable of these investigations these two approaches are combined along with subcellular targeting/analysis to measure Ca^2+^, PKC, CaMKII, and Annexin A4 translocation [8]. This is possible through the use of the calcium sensor Fura Red, a membrane targeted CFP-YFP based FRET sensor PM-CKAR, a cytosolic CFP-YFP based CamKII FRET sensor and an mOrange-mCherry based annexin A4 sensor. In another noteworthy investigation Ni et al developed a three color tandem sensor for cAMP and PKA activity, ICUEPID [34]. By combining a CFP/mCherry based PKA sensor, from CRY AKAR [35], with the molecular switch and acceptor molecule of Epac based cAMP sensor, AKAR2 [17].

As useful as these methods are, room for growth within the visible spectrum is limited. The method presented here makes much more efficient use of limited spectral range of fluorescent proteins. We have demonstrated the ability to monitor three spatially colocalized processes with only as many types fluorescent proteins. It should be possible develop novel constructs with additional, perhaps blue shifted, orange or far-red shifted, fluorescent proteins. Expansion of this method to four fluorescent proteins would allow six colocalized processes to be monitored simultaneously. Five fluorescent proteins would allow for monitoring ten processes and still would not use the entire visible spectrum. Especially when combined with the possibility of subcellular molecular targeting of sensors, experimental multiplexing may no longer be limited by orthogonality of fluorescent proteins or the finite size of the visible spectrum but more likely by the limits of sensor expression and the noise associated with measuring and separating overlapping fluorescent signals. Continued development of bright and photostable fluorescent proteins [10], [36] as well as engineering of FRET sensors to increase for increased dynamic range [37] will most certainly help address noise related issues and further the possibilities for FRET-based experimental multiplexing.

## Materials and Methods

### Recombinant DNA Procedures

In the development of the FRET sensors and tandem constructs mTurquoise2 [10], cpVenus variant 173 [11], and mCherry [12] were used but will be referred to simply as CFP, YFP, and RFP, respectively. To develop the CFP-15AA-YFP, CFP-15AA-RFP, and RFP-15AA-YFP tandem constructs, CFP and RFP were first cloned into the Clontech C1 vector between AgeI and BspEI. The sequence encoding the second fluorophore was then inserted between SalI and BamHI of these vectors resulting in the two FP sequences flanking the remaining multiple cloning site. These tandem constructs were used for calibration procedures as well as to generate the sensors. The sequences encoding the functional units of existing calcium, cAMP and PKA sensors (TN-XXL [16], Epac1(ΔDEP-CD) [18], and AKAR3 [17], [38]) were amplified by PCR. The functional units of TN-XXL and AKAR3 were inserted between BspEI and SalI, while that of Epac1 was inserted between EcoRI and SalI. All clones were verified by sequencing.

### Adherent Cell Culture, Transfection and Imaging

HeLa cells from the American Type Culture collection (ATCC) were grown in Dulbeccós modified Eaglés medium (DMEM) containing 10% fetal calf serum (FCS) and 1% penicillin/streptomycin at 37°C under 5% CO_2_. For transient transfection, cells were seeded at low-density on 18-mm #1.5 cover-slips (∼3×10^5^) and transfected using Lipofectamine 2000 Reagent (Invitrogen) according to the manufacturer’s instruction. Three hours after transfection, medium was exchanged and cells were serum starved over night before measurements. Before imaging cells are rinsed twice in Ringer’s solution (160 mM NaCl, 2.5 mM KCl, 2 mM CaCl2, 1 MgCl2 10 mM HEPES, 8 mM Glucose) and mounted in a custom imaging chamber. Cells are continuously superfused with gravity fed Ringer’s solution prior to agonist application. Solution exchange is performed by six channel valve control system (Warner Instruments, Hamden CT). Image stacks were acquired at 5 second intervals with 500 ms exposure with 430 nm excitation during CFP excitation, 300 ms exposure with 500 nm excitation during YFP excitation, and 500 ms exposure with 575 nm during RFP excitation. To activate the Gq signaling pathway and elicit calcium release from internal stores 10 uM histamine (Sigma, St. Louis, MO) was applied to the cells. To activate adenylate cyclase and inhibit phosphodiesterases, as to increase cAMP concentration and activate PKA 25 uM forskolin (Sigma, St. Louis, MO) and 100 uM IMBX (Sigma, St. Louis, MO) were applied to the cells. To increase intracellular calcium to saturate the response of calcium sensors 10 uM ionomycin (Sigma, St. Louis, MO) in ringers with 5 mM CaCl_2_ was applied to the cells.

### Fluorescence Spectroscopy

High resolution fluorescence spectra were measured using a Fluorolog-3 spectrofluorometer (HORIBA Jobin Yvon, München, Germany). Samples of detached cells expressing a given fluorescent protein were placed in 10-mm pathway quartz cuvettes (10×10 mm^2^) and continuously stirred by a magnetic stirrer. Excitation was provided and emission collected through 2 nm slits. The spectral contributions due to scattering and cellular autofluorescence were measured and subtracted from the fluorescence measurements.

### Fluorescence Microscopy

All images were acquired on a Nikon TE2000 inverted microscope (Nikon, Tokyo, Japan) with a 60× water-immersion objective (NA 1.2) controlled by Andor Iq software (Andor Technology, Belfast UK). Excitation was provided by Polychrome IV (Till photonics, Gräfelfing, Germany) at 430 nm for direct excitation of mTq2, 500 nm for direct excitation of cpVenus, and 575 nm for direct excitation of mCh using a Tripleband CFP/YFP/mCherry ET Filter Set (Chroma, Bellows Falls, VT, USA). Emission was split by a Quadview image splitter (Photometrics, Tucson, AZ, USA) equipped with 510LPXRXT, 560LPXR, and 600LPXR dichroic mirrors (Chroma, Bellows Falls, VT, USA) and projected onto an iXon Ultra EMCCD camera (Andor Technology, Belfast,UK).

### Image Acquisition, Processing and Analysis

Images were acquired and exported as raw 16-bit tiff files using Andor IQ software. Further analysis was performed in Matlab 2011a (Mathworks, Natick, MA). Spatial alignment was performed using a discrete Fourier transform (DFT) based sub-pixel image registration algorithm [39]. To quantify FRET time courses, 15×15 pixel (approximately 50 µm^2^) regions of interest in cytoplasm were selected and an excitation/emission spectra computed from the mean fluorescence intensities of the image stack for each time point. The total relative concentrations and scaled FRET efficiencies are then determined through non-negative linear unmixing of the sample spectrum with the reference and FRET spectra. To compute spatial maps of the total relative concentrations and FRET quantities per pixel linear unmixing and analysis was performed after 2×2 pixel binning.

The general workflow of a set of reference measurements, calibrations and FRET sample measurements is as follows:

1. **Reference sample measurements**. Acquire spectral images with excitation wavelength i over emission channels 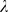 for samples selectively labeled with individual fluorescent species.
  - **Image processing.** For each spectral image:
    - Split dual or quad-view images into emission stacks
    - Correct for non-uniform field of illumination
    - Perform image registration of the entire ex./em. stack
    - Measure background from user defined ROI and subtract for each image
  - **Sample reference images.** Measure reference spectra.
    - Measure the mean fluorescence from a user defined ROI to compute 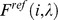 for each of the reference species.
2. **Compute the relative excitability terms**. Using the reference spectra from above along with values for quantum efficiency (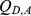) and unit normalized emission spectra sampled at 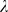 (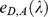), which can be measured or estimated from literature, compute the relative excitability term 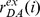
for each donor acceptor combination using,

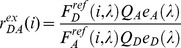
(10)(equivalent to equation 3 from [9]).

1. **Compute the FRET spectra.** Using the appropriate reference spectra and relative excitability terms from above compute the FRET spectra using,

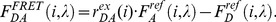
(11)

1. **Measure FRET sample.** Acquire a spectral image of the FRET sample with the same parameters under which the reference samples were measured. Perform the same image processing.

1. **Unmixing.** Using the measured reference spectra and the FRET spectra from above perform linear unmixing of the FRET sample spectra measured from a user defined ROI or on a per pixel basis for FRET imaging. Unmixing will return values representing a) the total relative concentration of the species in the sample relative to the corresponding reference concentration and b) values representing the FRET efficiency scaled by the concentration of donor-acceptor complexes relative to the corresponding donor reference concentration.
2. **Compute apparent FRET efficiencies**. By dividing the scaled FRET efficiency terms resulting from the unmixing performed above by the appropriate total relative concentration of donor or acceptor concentration the apparent FRET efficiencies, *Ef_D_* and *Ef_A_*, can be computed.

If additional information regarding the stoichiometry of expression of the fluorescent species in the sample is known it may be possible to determine an estimate for absolute FRET efficiency (scaled by some functional labeling probability, see [9]). This requires an additional set of calibration measurement.

- A1. **Known stoichiometry reference measurement.** Acquire spectral images of the known stoichiometry reference sample (e.g. tandem construct) with the same parameters under which the reference samples were measured. Perform the same image processing.
- A2. **Quantify the reference concentration ratio.** Perform the unmixing analysis outlined above and compute the total relative concentrations. Use these concentrations and the known stoichiometry of each tandem construct to compute the reference concentration ratio from equation 8 for each donor - acceptor pair in the sample.
- A3. **Determine sensor concentration.** Construct a set of linear equations analogous to equation 9 that define the known or estimated stoichiometry of the sensors in the sample. In this case, as in most, the stoichiometry of each sensor is 1∶1 so all coefficients are 1. Use the reference concentration ratios where appropriate to provide common denominators (i.e. [Y^T^]/[Y^ref^] = [CY]/[C^ref^] * R^TC^_CY_+[YR]/[Y^ref^], where R^TC^_CY_ = [C^ref^]/[Y^ref^]). Solve this set of equations to determine the relative sensor concentrations.
- A4. **Compute estimates for FRET efficiency**. By dividing the scaled FRET efficiency terms resulting from the unmixing performed in step 5 above by the appropriate relative sensor concentration the absolute FRET efficiencies can be estimated. It should be noted that interpretation of these values should still be performed with caution (see [9]).
